# Real-world safety profile of finerenone in patients with type 2 diabetes and chronic kidney disease: a multi-database pharmacovigilance analysis of the FAERS and JADER systems

**DOI:** 10.3389/fcdhc.2026.1984666

**Published:** 2026-10-20

**Authors:** Dan Zhang, Yun Zhao, Haiting Chen, Yang Miao

**Affiliations:** 1 Department of Pharmacy, Yancheng First Hospital Affiliated Hospital of Nanjing University Medical School, The First People’s Hospital of Yancheng, Yancheng, Jiangsu, China; 2 Oncology Department, Yancheng First Hospital Affiliated Hospital of Nanjing University Medical School, The First People’s Hospital of Yancheng, Yancheng, Jiangsu, China

**Keywords:** adverse drug reactions, FAERS, finerenone, JADER, pharmacovigilance, signal detection

## Abstract

**Background:**

Finerenone, a novel nonsteroidal mineralocorticoid receptor antagonist, has been shown to lower the risk of renal and cardiovascular events in patients with type 2 diabetes mellitus (T2DM) and chronic kidney disease (CKD). However, real-world safety data beyond clinical trials remain limited, particularly regarding the spectrum and timing of adverse events (AEs) in routine clinical practice.

**Methods:**

Disproportionality analyses (Reporting Odds Ratio and Proportional Reporting Ratio) were performed on finerenone-associated AE reports from the U.S. Food and Drug Administration (FDA) Adverse Event Reporting System (FAERS, Q3 2021-Q1 2025) and the Japanese Adverse Drug Event Report (JADER) database, following data deduplication and the Medical Dictionary for Regulatory Activities (MedDRA, version 27.1) standardization. The time-to-onset (TTO) of AEs was also analyzed.

**Results:**

A total of 1,545 (FAERS) and 210 (JADER) reports with finerenone as the primary suspect were identified. Consistent with its known profile, hyperkalemia, increased blood creatinine, decreased glomerular filtration rate (GFR), and renal impairment generated significant signals in both databases. Notably, the analysis revealed several potential new signals that have not been emphasized in the drug label, including increased urine albumin/creatinine ratio (UACR), abnormal hepatic function, penile gangrene, and dizziness. The median TTO for AEs was 36 days (FAERS) and 51 days (JADER), with a substantial proportion (45.16% in FAERS, 60.18% in JADER) occurring within the first two months of treatment.

**Conclusions:**

This bilateral pharmacovigilance analysis confirms known risks of finerenone and identifies several potential new AE signals in patients with T2DM-CKD. The TTO analysis underscores the need for enhanced monitoring during the initial two months of therapy. This study generates actionable evidence to optimize risk management strategies and inform safer clinical use of finerenone in this high-risk population.

## 1 Introduction

Diabetes nephropathy is a serious microvascular complication of diabetes, which is the main cause of end-stage renal disease and chronic renal disease worldwide (1–3). For patients with type 2 diabetes mellitus (T2DM) or chronic kidney disease (CKD), it is important to prevent kidney function decline (4). Finerenone, a novel nonsteroidal mineralocorticoid receptor antagonist, is approved for the management of CKD in patients with T2DM (5). It was first marketed in the United States in July 2021. Recent studies have demonstrated its efficacy in heart failure with mildly reduced (HFmrEF) or preserved (HFpEF) ejection fraction (6, 7). Finerenone ameliorates mitochondrial dysfunction in diabetic tubular nephropathy via the PI3K/Akt/eNOS pathway, as well as reduces renal and cardiovascular events through anti-inflammatory and anti-fibrotic mechanisms (8–10). Although pivotal clinical trials have confirmed its therapeutic benefits, they have also reported adverse reactions including hyperkalemia and renal impairment (10, 11). However, randomized controlled trials (RCTs) operate under stringent inclusion criteria and typically feature limited sample sizes. Consequently, they may not fully capture the spectrum of rare or long-term adverse events that emerge in real-world clinical practice.

Spontaneous reporting systems, for example, the Japanese Adverse Drug Event Report database (JADER) and the US Food and Drug Administration (FDA) Adverse Event Reporting System (FAERS) can complement the limitations of RCTs by enabling the identification of adverse safety signals through large-scale data mining. Recently, several studies have begun to explore finerenone’s safety profile using real-world data. For example, Jin et al. (12) and Huang et al. (13) conducted disproportionality analyses of finerenone-associated adverse events using the FAERS database, confirming known risks such as hyperkalemia while also identifying potential new signals. However, these studies were limited to a single database (FAERS) and relatively short follow-up periods.

Therefore, a comprehensive pharmacovigilance analysis of finerenone was performed in this study, aiming to jointly mine and compare safety signals from both the FAERS and JADER databases, thereby providing a more comprehensive evidence base for optimizing its clinical safety monitoring strategies.

## 2 Methods

### 2.1 Data sources and processing

Data sourced from the FAERS database on adverse drug reactions (ADRs) related to finerenone since its market approval (Q3–2021 to Q1 2025) were downloaded. ASCII-formatted data packages were imported into R (version 4.4.2) for integration, cleaning, and standardization. First, seven standardized tables—DEMO (demographics), DRUG (drug), REAC (reactions), OUTC (outcomes), RPSR (report sources), THER (therapy), and INDI (indications) from each quarter were vertically merged by field names to construct a complete time-series dataset. Data standardization included unifying missing value encodings (e.g., converting blanks, NA, NULL, etc., to standardized NA) and normalizing drug names (e.g., unifying variants such as “FINERENONE” and “FINERENONUM” to a standard nomenclature).

Subsequently, data deduplication was performed according to the FDA’s “FAERS Data Removal Guide.” Duplicate cases in the DEMO table were identified based on primaryid and caseid, retaining the latest version (using the fda_dt field). The deduplicated DEMO table was then mapped to the other six tables via the primaryid field, employing a cascading deletion strategy to ensure data consistency. The effectiveness of deduplication was verified by comparing record counts before and after the process, and the completeness of key fields (e.g., age, gender, report date, ADR outcomes) was examined to ensure data quality.

After excluding reports with duplicates, misspelled drug names, and irrelevant adverse events, all adverse events were coded using Preferred Terms (PT) from MedDRA (version 27.1) and categorized by System Organ Class (SOC). Possible drug names for finerenone were collected from authoritative sources such as Drugs@FDA. The role_cod field in the DRUG table was restricted to “PS” (primary suspect) to retrieve relevant ADR reports for finerenone from the cleaned dataset.

ADR data for finerenone since its approval were also downloaded from the JADER database. Data were loaded into R (version 4.4.2), duplicate entries across JADER’s four core tables were removed. Reports related to finerenone were extracted, and adverse events were coded with PTs from MedDRA (version 27.1).

### 2.2 Signaling methods

Data mining algorithms, including the Reporting Odds Ratio (ROR), Proportional Reporting Ratio (PRR), and the Medicines and Healthcare products Regulatory Agency (MHRA) comprehensive criteria, were employed for signal detection. Specific algorithms and thresholds were based on established references (14, 15).

In the ROR method, a signal was considered significant if the adverse event (AE) count was ≥3 and the lower limit of 95% confidence interval (CI) exceeded 1. For the PRR method, a signal was deemed valid when the AE count was ≥3, the PRR value was ≥2, and the χ² value was ≥4. Elevated PRR and ROR values directly indicated a stronger drug-event association signal.

### 2.3 Time-to-onset analysis

We calculated the time interval between the start of treatment with finerenone (START_DT) and the onset of adverse events (EVENT_DT). Reports containing errors or missing dates are excluded. The median was used to describe the time to onset.

## 3 Results

### 3.1 Basic characteristics of finerenone adverse event reports

We identified 1,545 AE reports in FAERS that listed finerenone as the primary suspected drug following data processing. Males accounted for 14.36% more reports than females. The majority of reports were from elderly patients aged 65–85 years. The United States submitted the most reports (70.94%), followed by Japan, China, Israel, and Germany. The most common indications for finerenone use were CKD and T2DM.

The JADER database identified 210 AE reports with finerenone as the primary suspect. Males represented 39.52% more reports than females. Most reports involved patients aged ≥70 years. The main indications included T2DM complicated with CKD and diabetic kidney disease (**Table 1**).

**Table 1:** Characteristics of finerenone-associated reports.

Factors	FAERS	JADER
	Number of events(%)	Number of events(%)
Gender		
Male	738(47.76)	128(60.95)
Female	516(33.40)	45(21.43)
Unknow	291(18.83)	37(17.62)
Age		
	18-64 200(12.94)	20-69 44(20.95)
	65-85 541(35.02)	≥70 95(45.24)
	>85 54(3.50)	Unknow 71(33.81)
	Unknow 750(48.54)	
Reported countries (top five)		
America	1096(70.94)	
Japan	137(8.87)	210(100.00)
China	56(3.62)	
Israel	26(1.68)	
Germany	24(1.55)	
Indication(top three)		
	chronic kidney disease 347(33.14)	diabetic kidney disease 70(28.57)
	type 2 diabetes mellitus 232(22.16)	chronic kidney disease in type 2 diabetes 69(28.16)
	diabetic peripheral neuropathy 96(9.17)	chronic kidney disease 8(3.27)
Report year		
2021	51(3.30)	
2022	363(23.50)	18(8.57)
2023	468(30.29)	64(30.48)
2024	496(32.10)	128(60.95)
2025	167(10.81)	

AE reports were significantly higher in males than in females across both databases and were mainly reported in the elderly. The reported indications were consistent with the approved label, primarily related to CKD associated with type 2 diabetes. The number of AE reports peaked in 2024 across both databases.

### 3.2 System organ class classification of AE signals

After data processing, a total of 565 preferred terms (PTs) meeting the predefined thresholds were identified as signals in FAERS, corresponding to 2,777 reported cases. These adverse drug events (ADEs) were classified into 26 SOC categories. The most frequently affected categories included various investigations, general disorders and administration site conditions, metabolism and nutrition disorders, renal and urinary disorders, and gastrointestinal system disorders. Among these, “various investigations” was the most common, accounting for 27.12% of all reports.

The JADER database yielded 52 significant PT signals, encompassing 242 reports. These ADEs were categorized into 18 SOCs, with the most prominent being metabolism and nutrition disorders, renal and urinary disorders, various investigations, nervous system disorders. Metabolism and nutrition disorders represented the highest proportion at 51.24%.

Both databases indicated that the primary SOCs associated with finerenone were metabolism and nutrition disorders, renal and urinary disorders, various investigations (**Figure 1**).

**Figure 1:**
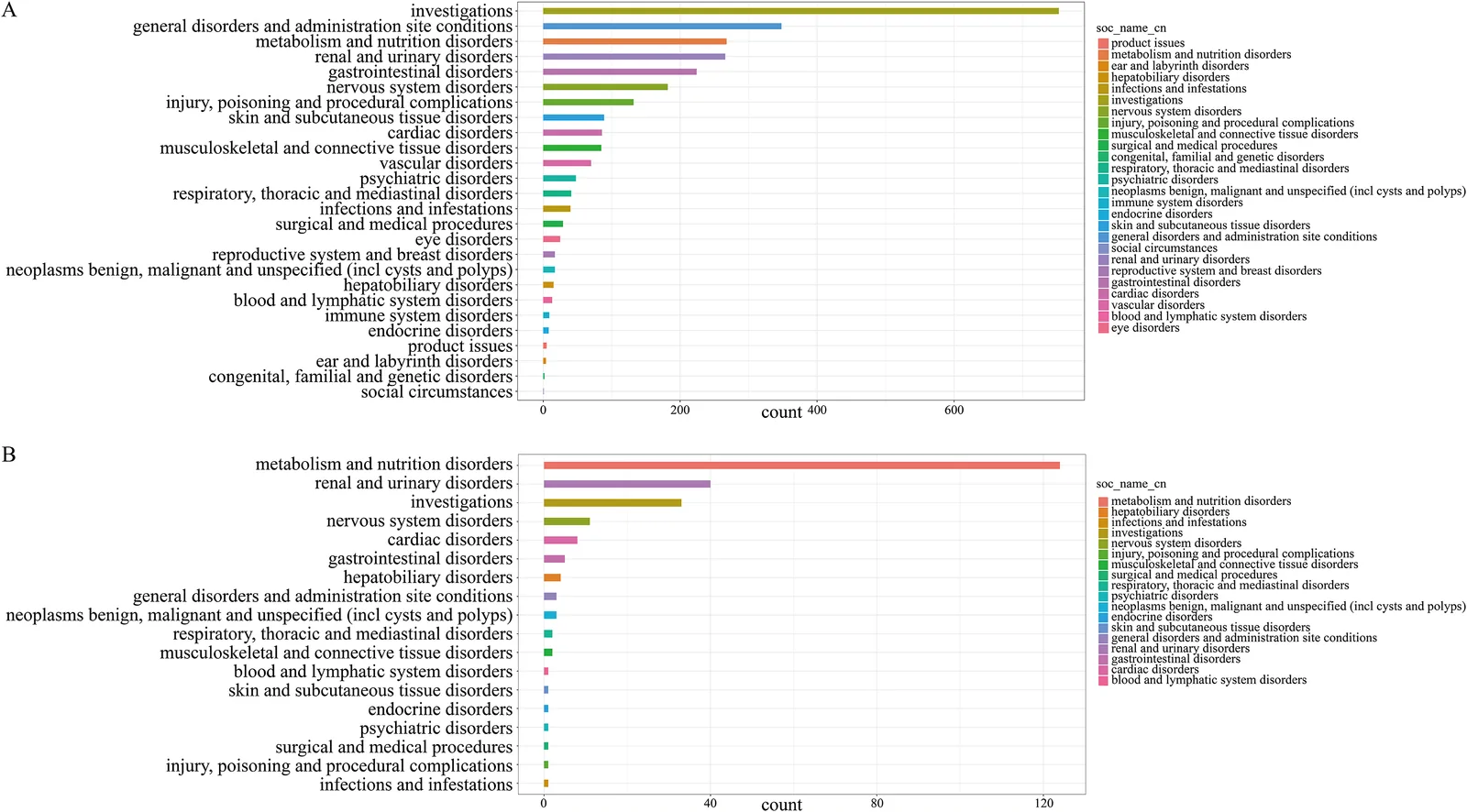
SOC classification of PT for finerenone ADE. **(A)** Data sourced from FAERS database. **(B)** Data sourced from JADER database.

### 3.3 Analysis of adverse event signals for finerenone

The detected Preferred Term (PT) signals were ranked by signal strength (ROR value) and frequency, and the top 15 PT signals from FAERS and JADER are presented in **Supplementary Tables S1** and **S2** and visualized in **Figures 2** and **3**, respectively.

**Figure 2:**
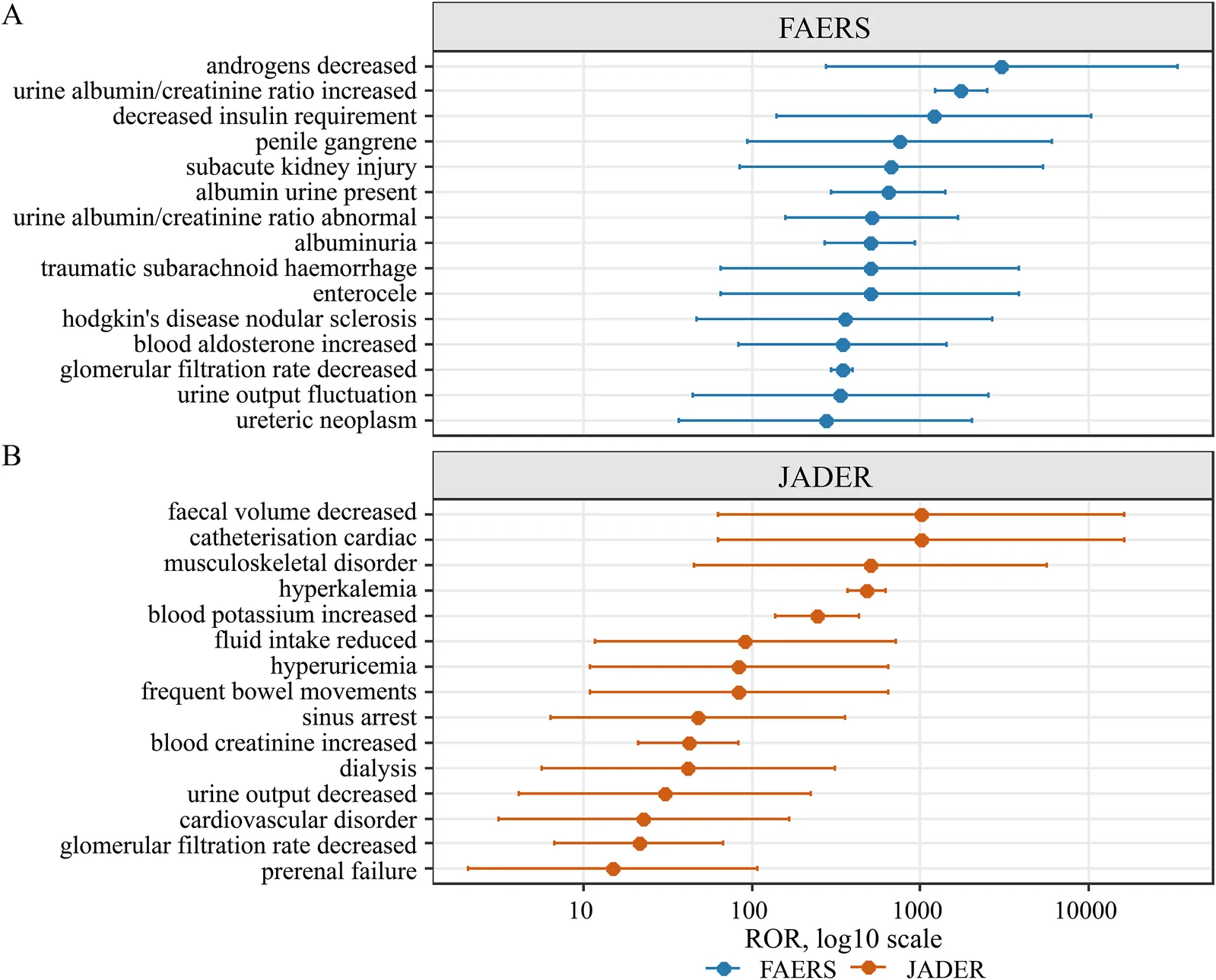
Top 15 finerenone-related PT signals by reporting odds ratio (ROR) with 95% confidence interval (CI). **(A)** Data sourced from FAERS database. **(B)** Data sourced from JADER database. The x-axis is shown on a log10 scale. Decreased UACR met the disproportionality threshold but represents an intended pharmacodynamic effect of finerenone rather than a true adverse reaction; it was therefore excluded from the main signal figure.

**Figure 3:**
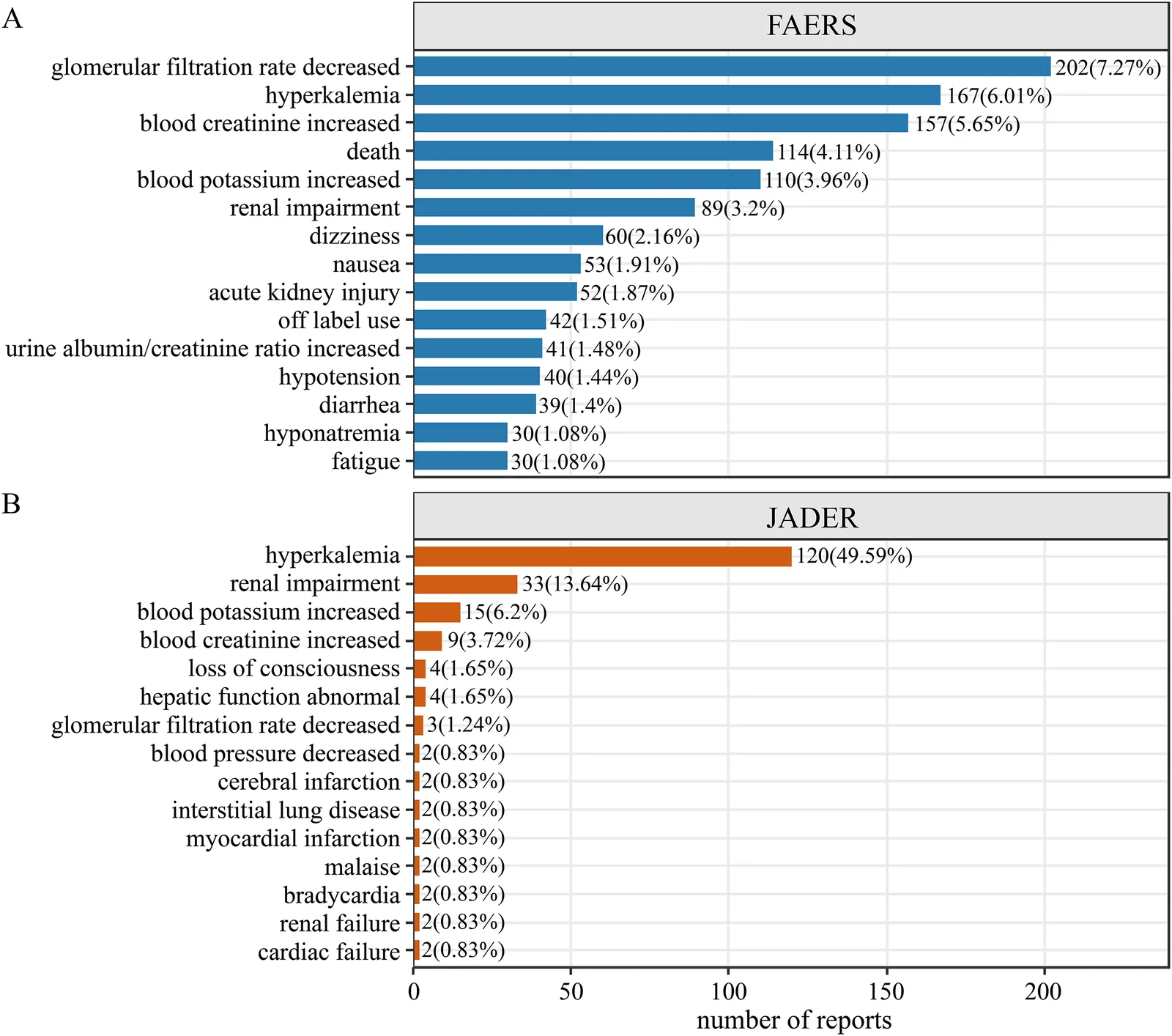
Top 15 most frequent finerenone-related PT signals. **(A)** Data sourced from FAERS database. **(B)** Data sourced from JADER database.

In FAERS, adverse events (AEs) with the highest signal strength were primarily related to various investigations, renal and urinary disorders, metabolism and nutrition disorders. Among them, decreased UACR met the disproportionality threshold but is a recognized pharmacodynamic effect of finerenone rather than a true adverse reaction; therefore, it was not considered a novel AE signal. After excluding recognized pharmacodynamic effects, the leading candidate signals were decreased androgens, increased UACR, decreased insulin requirement, penile gangrene, and subacute kidney injury. The most frequently reported AEs fell under the categories of various investigations, metabolism and nutrition disorders, general disorders and administration site conditions. The top five AEs by frequency were: decreased glomerular filtration rate, hyperkalemia, increased blood creatinine, death, and increased blood potassium.

In JADER, the AEs with the strongest signals were mainly associated with various investigations and metabolism and nutrition disorders. The top five AEs by signal strength were: decreased faeces quantity, cardiac catheterization, musculoskeletal disorders, hyperkalemia, and increased blood potassium. The most common AEs by frequency pertained to metabolism and nutrition disorders, various investigations, renal and urinary disorders. The top five high-frequency AEs were: hyperkalemia, renal impairment, increased blood potassium, increased blood creatinine, and loss of consciousness.

Hyperkalemia, increased blood potassium, increased blood creatinine, decreased glomerular filtration rate, and renal impairment were among the AEs frequently reported in both databases.

After consolidating and analyzing the AE signals, and excluding categories such as social circumstances and product issues, a comparison with the drug label identified several potential new adverse reactions, including dizziness, increased UACR, abnormal hepatic function, and penile gangrene. In addition, nausea, diarrhea, hypotension, and acute kidney injury showed signals in one or both databases; however, these were considered secondary or supportive signals and require further validation. Renal impairment was also detected but has been documented in related clinical trials. Although decreased UACR met the statistical threshold for a disproportionality signal in FAERS, it is a recognized pharmacodynamic effect of finerenone rather than a true adverse reaction. It is therefore reported here as a laboratory change/signal and is not interpreted as a novel safety signal.

### 3.4 Time-to-onset of finerenone-associated adverse events

The FAERS database contained 186 reports detailing finerenone-associated adverse events, with a median time to onset of 36 days. The JADER database has a total of 108 reports that provide detailed records of the time to onset of finerenone-related adverse events, with a median duration of 51 days (**Figure 4**). Most adverse events of both occurred in the first month (FAERS, 84, 45.16%; JADER, 34, 31.48%).

**Figure 4:**
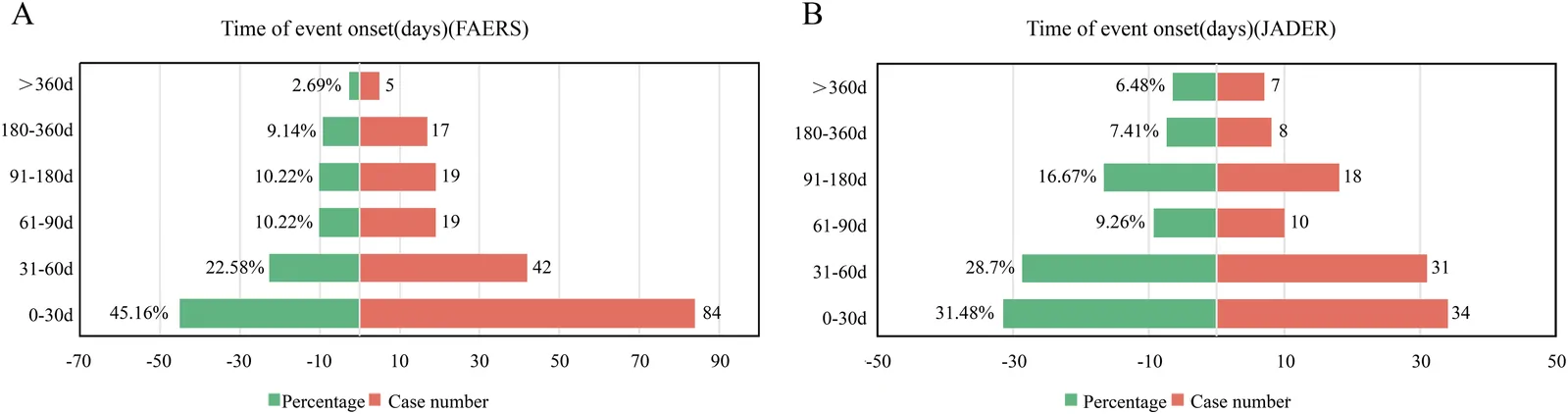
Time to onset of adverse events associated with finerenone. **(A)** Data sourced from FAERS database. **(B)** Data sourced from JADER database.

## 4 Discussion

The novel non-steroidal mineralocorticoid receptor antagonist finerenone is indicated for patients with CKD associated with type 2 diabetes, reducing their risk of renal and cardiovascular events including kidney failure and death. However, its real-world safety profile remains to be fully elucidated. The present study comprehensively assessed finerenone-related adverse reaction signals using standardized data mining methods based on the JADER and FAERS databases.

As for demographic characteristics, our findings revealed significant gender and age disparities in finerenone-related adverse events. Male patients accounted for a notably higher proportion of reports than females (FAERS: 47.76% vs. 33.40%; JADER: 60.95% vs. 21.43%). Elderly patients constituted the majority of reported cases, with the highest proportion observed in the 65–85 age group in FAERS (35.02%) and in patients aged ≥70 years in JADER (45.24%). These findings are consistent with the epidemiological characteristics of CKD and type 2 diabetes populations (16). No patients under 18 years of age receiving finerenone were reported, consistent with its approved use in adults. Special attention should be paid to adverse reactions in elderly patients due to their higher burden of comorbidities, which may exacerbate clinical outcomes.

At the System Organ Class (SOC) level, finerenone-associated adverse events primarily involved metabolism and nutrition disorders, renal and urinary disorders, various investigations, which is largely consistent with the four SOC categories indicated in the drug label. Frequency analysis showed that decreased glomerular filtration rate (GFR), hyperkalemia, increased blood creatinine, and elevated blood potassium were frequently reported in both databases, corroborating findings from the drug label and previous studies (12, 13). Notably, decreased GFR is an independent risk factor for all-cause and cardiovascular mortality (17). However, in the context of treating CKD and hypertension, an initial transient decline in estimated GFR (eGFR) differs significantly from a sustained reduction. Studies have shown that finerenone rapidly affects renal hemodynamics shortly after initiation of treatment, leading to a noticeable eGFR decrease within the first month. This decline is generally self-limiting and non-progressive, and may even confer long-term renal benefits (18). Changes in serum creatinine levels largely reflect glomerular filtration capacity, and reduced GFR is a common feature of disease progression in type 2 diabetes. Therefore, the observed GFR reduction may be attributable to disease progression rather than drug effects. Patients with CKD and type 2 diabetes are at increased risk of hyperkalemia due to impaired renal function (19). Given the high incidence of hyperkalemia associated with finerenone, close monitoring of serum potassium levels is mandated before and during treatment.

Our analysis revealed four main candidate new signals: increased UACR, abnormal hepatic function, penile gangrene, and dizziness. In addition, several secondary signals of interest were observed, including nausea, diarrhea, hypotension, and acute kidney injury. These were reported less consistently or have been documented in clinical trials, and therefore should be regarded as supportive findings rather than confirmed novel adverse reactions. Although these events were reported infrequently, they demonstrated significant signal strength and warrant clinical attention. The consistency of these signals across two independent databases (FAERS and JADER) strengthens their potential validity. Renal impairment and acute kidney injury have also been documented in related clinical trials (10, 11). The paradoxical occurrence of renal events despite the drug’s nephroprotective indications could be attributed to the hemodynamic effects of MR blockade, disease progression in this high-risk population, or interactions with concomitant medications such as sodium-glucose cotransporter 2 (SGLT2) inhibitors or renin-angiotensin-aldosterone system (RAAS) inhibitors, necessitating further investigation. Symptoms such as dizziness, nausea, and diarrhea may be associated with finerenone-induced hypotension and hyponatremia. Penile gangrene, a rare but serious event, might be linked to finerenone’s potential effects on vascular tone and perfusion, particularly in patients with pre-existing severe vascular disease, a common comorbidity in the target population. The impact on liver function may be linked to its metabolism primarily via CYP3A4 (20), though the exact mechanisms require further study. The signal for increased UACR, detected with high strength in FAERS, is particularly intriguing. While initially counterintuitive for a renoprotective agent, this might reflect a hemodynamic adjustment or could be an early marker of a specific drug-induced tubular effect, meriting dedicated investigation. In contrast, decreased UACR is an intended pharmacodynamic effect and should not be regarded as an adverse reaction.

Notably, we observed both consistencies and disparities between the FAERS and JADER databases. Hyperkalemia was the dominant signal in both, but it accounted for a substantially higher proportion of reports in JADER (49.59% of significant PTs) compared to FAERS (6.01%). This discrepancy could stem from differences in patient characteristics (e.g., diet, genetics), clinical practice patterns (e.g., monitoring intensity, concomitant drug use), or reporting behaviors between the two countries. The median time for adverse events to occur in this study was 36 days or 51 days, respectively. Nearly half of the adverse events in FAERS occurred in the first month of treatment (45.16%), while adverse events in JADER mainly occurred in the first two months of treatment (60.18%), indicating the importance of early monitoring.

The strengths of this study include: 1) a systematic analysis of finerenone’s safety profile using two large spontaneous reporting databases (JADER and FAERS); 2) the application of internationally recognized disproportionality analysis methods (ROR and PRR), ensuring reliable results; and 3) a relatively large sample size (1,545 cases from FAERS and 210 from JADER), enhancing the robustness of the conclusions. However, several caveats should be noted: 1) underreporting and reporting bias are intrinsic limitations of spontaneous reporting systems; 2) consumers can also submit reports, which may lack professional accuracy; 3) causality between drug and event is not required for submission, so some reported associations may be coincidental; and 4) confounding factors such as concomitant medications and underlying diseases were not fully controlled for in either database.

Although spontaneous reporting systems such as FAERS and JADER are valuable for post-marketing safety surveillance, disproportionality signals should be interpreted cautiously. A recent review of new ADR signals identified between 2017 and 2021 found that none of the 56 suspected new ADRs from 24 drugs had undergone detailed confirmatory evaluation in subsequent literature, despite the original studies having been cited a median of seven times (21). This finding highlights that most signals generated by disproportionality analyses remain unverified as either genuine ADRs or false alarms. Consistent with this, the novel signals identified in our study—including increased UACR, abnormal hepatic function, penile gangrene, and dizziness—should be regarded as hypothesis-generating rather than confirmatory. Future studies incorporating prospective cohorts, electronic health records, or other causal inference approaches are needed to validate the clinical significance of these signals.

Based on our findings, we propose the following clinical recommendations: 1) enhance monitoring of serum potassium, liver function, and renal function (including urinalysis for albumin/creatinine ratio) during finerenone therapy, especially within the first 2 months; 2) conduct targeted studies (e.g., pharmacokinetic, pharmacogenomic) to clarify causal relationships for newly identified signals such as abnormal hepatic function and increased UACR; and 3) develop individualized treatment monitoring plans, considering patient-specific factors like age, comorbidities (especially cardiovascular disease), and concomitant medications. Future research incorporating electronic health records (EHR) or prospective cohort designs is encouraged to further validate the clinical significance of these signals. In conclusion, the use of finerenone in clinical practice should be accompanied by personalized monitoring strategies tailored to patient characteristics. This study provides critical evidence to inform finerenone’s risk management and safe application in clinical practice.

## 5 Conclusions

This bilateral pharmacovigilance analysis confirms the known renal and metabolic risks of finerenone and identifies several potential new safety signals-including increased UACR, abnormal hepatic function, penile gangrene, and dizziness-in patients with T2DM-CKD. The time-to-onset analysis reveals that a substantial proportion of adverse events occur within the first two months of treatment (median: 36–51 days), highlighting a critical window for enhanced monitoring. While these signals are hypothesis-generating and require prospective validation, our findings provide actionable evidence to guide risk stratification and individualized monitoring strategies, particularly for serum potassium, renal function, and liver enzymes during early therapy. Clinicians should remain vigilant for both established and emerging adverse events to optimize the safe use of finerenone in this high-risk population.

## Data Availability

The original contributions presented in the study are included in the article/**Supplementary Material**. Further inquiries can be directed to the corresponding author.

## References

[1] Gupta S Dominguez M Golestaneh L . Diabetic kidney disease: An update. In: The Medical Clinics of North America (2023). Philadelphia, PA: Elsevier. 10.1016/j.mcna.2023.03.004 37258007

[2] Zheng L Chen W Yao K Lin Y Liao C Zhou T . Mechanism of mesenchymal stem cells in treating diabetic kidney disease. Stem Cell Res Ther. (2025) 16:629. doi: 10.1186/s13287-025-04750-3 41220025 PMC12606926

[3] Młynarska E Buławska D Czarnik W Hajdys J Majchrowicz G Prusinowski F . Novel insights into diabetic kidney disease. Int J Mol Sci. (2024) 25:10222. doi: 10.3390/ijms251810222 39337706 PMC11432709

[4] Doshi SM Friedman AN . Diagnosis and management of type 2 diabetic kidney disease. Clin J Am Soc Nephrol. (2017) 12(8):CJN.11111016. doi: 10.2215/cjn.11111016 28280116 PMC5544517

[5] Frampton JE . Finerenone: first approval. Drugs. (2021) 81:1787–94. doi: 10.1007/s40265-021-01599-7 34519996

[6] Solomon SD McMurray JJV Vaduganathan M Claggett B Jhund PS Desai AS . Finerenone in heart failure with mildly reduced or preserved ejection fraction. N Engl J Med. (2024) 391:1475–85. doi: 10.1056/NEJMoa2407107 39225278

[7] Vaduganathan M Claggett BL Lam CSP Pitt B Senni M Shah SJ . Finerenone in patients with heart failure with mildly reduced or preserved ejection fraction: rationale and design of the FINEARTS-HF trial. Eur J Heart Fail. (2024) 26:1324–33. doi: 10.1002/ejhf.3253 38742248

[8] Yao L Liang X Liu Y Li B Hong M Wang X . Non-steroidal mineralocorticoid receptor antagonist finerenone ameliorates mitochondrial dysfunction via PI3K/Akt/eNOS signaling pathway in diabetic tubulopathy. Redox Biol. (2023) 68:102946. doi: 10.1016/j.redox.2023.102946 37924663 PMC10661120

[9] Grune J Beyhoff N Smeir E Chudek R Blumrich A Ban Z . Selective mineralocorticoid receptor cofactor modulation as molecular basis for finerenone's antifibrotic activity. Hypertension. (2018) 71:599–608. doi: 10.1161/hypertensionaha.117.10360 29437893

[10] Bakris GL Agarwal R Anker SD Pitt B Ruilope LM Rossing P . Effect of finerenone on chronic kidney disease outcomes in type 2 diabetes. N Engl J Med. (2020) 383:2219–29. doi: 10.1056/NEJMoa2025845 33264825

[11] Rossing P Anker SD Filippatos G Pitt B Ruilope LM Birkenfeld AL . Finerenone in patients with chronic kidney disease and type 2 diabetes by sodium-glucose cotransporter 2 inhibitor treatment: the FIDELITY analysis. Diabetes Care. (2022) 45:2991–8. doi: 10.2337/dc22-0294 35972218 PMC9862372

[12] Jin Y Fan M Zheng X Zhu S . Post-marketing safety of finerenone: a disproportionality analysis of the FDA adverse event reporting system. Expert Opin Drug Saf. (2025) 24(9):1047–52. doi: 10.1080/14740338.2024.2392006 39167069

[13] Huang Y Gao H Lin Y Chen X Chen M Chen M . Real-world pharmacovigilance study of FDA adverse event reporting system events for finerenone. Expert Opin Drug Saf. (2025) 24(12):1479–86. doi: 10.1080/14740338.2024.2412218 39385540

[14] Sakaeda T Tamon A Kadoyama K Okuno Y . Data mining of the public version of the FDA adverse event reporting system. Int J Med Sci. (2013) 10:796–803. doi: 10.7150/ijms.6048 23794943 PMC3689877

[15] Gu J Qu Y Shen Y Zhou Q Jiang Y Zhu H . Comprehensive analysis of adverse events associated with pimavanserin using the FAERS database. J Affect Disord. (2024) 362:742–8. doi: 10.1016/j.jad.2024.07.103 39029673

[16] Agarwal R Filippatos G Pitt B Anker SD Rossing P Joseph A . Cardiovascular and kidney outcomes with finerenone in patients with type 2 diabetes and chronic kidney disease: the FIDELITY pooled analysis. Eur Heart J. (2022) 43:474–84. doi: 10.1093/eurheartj/ehab777 35023547 PMC8830527

[17] Gregg LP Tio MC Li X Adams-Huet B de Lemos JA Hedayati SS . Association of monocyte chemoattractant protein-1 with death and atherosclerotic events in chronic kidney disease. Am J Nephrol. (2018) 47:395–405. doi: 10.1159/000488806 29874658 PMC6145464

[18] Sato A . Does the temporary decrease in the estimated glomerular filtration rate (eGFR) after initiation of mineralocorticoid receptor (MR) antagonist treatment lead to a long-term renal protective effect? Hypertens Res. (2019) 42:1841–7. doi: 10.1038/s41440-019-0320-9 31492909

[19] Bem D Sugrue D Wilding B Zile I Butler K Booth D . The effect of hyperkalemia and long inter-dialytic interval on morbidity and mortality in patients receiving hemodialysis: a systematic review. Ren Fail. (2021) 43:241–54. doi: 10.1080/0886022x.2020.1871012 33478329 PMC7833048

[20] Heinig R Eissing T . The pharmacokinetics of the nonsteroidal mineralocorticoid receptor antagonist finerenone. Clin Pharmacokinet. (2023) 62:1673–93. doi: 10.1007/s40262-023-01312-9 37875671 PMC10684710

[21] Loke YK Mattishent K Navaneetharaja N . New adverse drug reaction signals from 2017 to 2021-genuine alerts or false alarms? Pharm (Basel). (2024) 2024:12. doi: 10.3390/pharmacy12010033 38392940 PMC10892108

