# Supplementary material for: Real-world safety profile of finerenone in patients with type 2 diabetes and chronic kidney disease: a multi-database pharmacovigilance analysis of the FAERS and JADER systems
Source: Front Clin Diabetes Healthc. 2026 Oct 20;7:1984666. doi: 10.3389/fcdhc.2026.1984666 (PMC13727449; doi:10.3389/fcdhc.2026.1984666)
Supplement: Supplementary file 1 [file SupplementaryFile1.docx]

# Supplementary Table S1. Top 15 finerenone AE signals by strength

| PT | SOC | Case(%） | ROR(95%CI） | PRR(χ2） |
| --- | --- | --- | --- | --- |
| **FAERS** |  |  |  |  |
| androgens decreased | investigations | 1(0.04) | 3009.86(272.83,33204.35) | 3008.77(2004.52) |
| urine albumin/creatinine ratio increased | investigations | 41(1.48) | 1738.99(1226.71,2465.2) | 1713.33(54615.16) |
| decreased insulin requirement | metabolism and nutrition disorders | 1(0.04) | 1203.94(140.61,10308.78) | 1203.51(1001.26) |
| penile gangrene | infections and infestations | 1(0.04) | 752.46(94.08,6018.42) | 752.19(666.84) |
| subacute kidney injury | renal and urinary disorders | 1(0.04) | 668.86(84.71,5281.29) | 668.62(599.96) |
| albumin urine present | investigations | 7(0.25) | 639.84(293.31,1395.78) | 638.22(4026.55) |
| urine albumin/creatinine ratio abnormal | investigations | 3(0.11) | 516.35(158.71,1679.87) | 515.79(1419.69) |
| albuminuria | renal and urinary disorders | 11(0.40) | 503.45(271.85,932.35) | 501.46(5071.53) |
| traumatic subarachnoid haemorrhage | injury, poisoning and procedural complications | 1(0.04) | 501.64(65.2,3859.37) | 501.46(461.04) |
| enterocele | gastrointestinal disorders | 1(0.04) | 501.64(65.2,3859.37) | 501.46(461.04) |
| hodgkin's disease nodular sclerosis | neoplasms benign, malignant and unspecified (incl cysts and polyps) | 1(0.04) | 354.1(47.11,2661.77) | 353.97(332.42) |
| blood aldosterone increased | investigations | 2(0.07) | 344.11(82.72,1431.41) | 343.86(646.77) |
| glomerular filtration rate decreased | investigations | 202(7.27) | 343(296.2,397.19) | 318.12(60665.55) |
| urine output fluctuation | investigations | 1(0.04) | 334.43(44.63,2506.08) | 334.31(314.82) |
| ureteric neoplasm | neoplasms benign, malignant and unspecified (incl cysts and polyps) | 1(0.04) | 273.62 ( 36.87 - 2030.72 ) | 273.52 ( 259.72 ) |
| **JADER** |  |  |  |  |
| faecal volume decreased | investigations | 1(0.41) | 1006.49(62.77,16138.51) | 1002.34(500.17) |
| catheterisation cardiac | investigations | 1(0.41) | 1006.49(62.77,16138.51) | 1002.34(500.17) |
| musculoskeletal disorder | musculoskeletal and connective tissue disorders | 1(0.41) | 503.24(45.48,5568.59) | 501.17(332.79) |
| hyperkalemia | metabolism and nutrition disorders | 120(49.59) | 478.11(366.12,624.37) | 241.53(23221.57) |
| blood potassium increased | investigations | 15(6.20) | 242.79(136.55,431.71) | 227.8(2760.8) |
| fluid intake reduced | metabolism and nutrition disorders | 1(0.41) | 91.5(11.77,711.47) | 91.12(81.71) |
| frequent bowel movements | gastrointestinal disorders | 1(0.41) | 83.87(10.86,647.56) | 83.53(75.27) |
| hyperuricemia | investigations | 1(0.41) | 83.87(10.86,647.56) | 83.53(75.27) |
| sinus arrest | cardiac disorders | 1(0.41) | 47.92(6.42,357.71) | 47.73(43.68) |
| blood creatinine increased | investigations | 9(3.72) | 41.98(21.29,82.75) | 40.45(333.18) |
| dialysis | surgical and medical procedures | 1(0.41) | 41.93(5.65,311.21) | 41.76(38.2) |
| urine output decreased | investigations | 1(0.41) | 30.5(4.15,223.87) | 30.37(27.58) |
| cardiovascular disorder | cardiac disorders | 1(0.41) | 22.87(3.14,166.67) | 22.78(20.37) |
| glomerular filtration rate decreased | investigations | 3(1.24) | 21.43(6.78,67.71) | 21.18(56.51) |
| prerenal failure | renal and urinary disorders | 1(0.41) | 15.02(2.08,108.62) | 14.96(12.84) |

Abbreviations: Decreased UACR met the pre-specified disproportionality threshold but is an intended pharmacodynamic effect of finerenone rather than a true adverse reaction. It was therefore excluded from the main AE signal analysis, and the next ranked PT signal was included to maintain the top 15 signals.

# Supplementary Table S2. 15 most frequent AE signals associated with finerenone

| PT | SOC | Case(%) | ROR(95%CI) | PRR(χ2) |
| --- | --- | --- | --- | --- |
| **FAERS** |  |  |  |  |
| glomerular filtration rate decreased | investigations | 202(7.27) | 343(296.2,397.19) | 318.12(60665.55) |
| hyperkalemia | metabolism and nutrition disorders | 167(6.01) | 145.33(124.08,170.22) | 136.65(21998.48) |
| blood creatinine increased | investigations | 157(5.65) | 67.84(57.7,79.76) | 64.06(9651.8) |
| death | general disorders and administration site conditions | 114(4.11) | 3.09(2.56,3.72) | 3(154.24) |
| blood potassium increased | investigations | 110(3.96) | 207.31(170.8,251.62) | 199.14(20995.25) |
| renal impairment | renal and urinary disorders | 89(3.20) | 22.93(18.56,28.34) | 22.23(1800.42) |
| dizziness | nervous system disorders | 60(2.16) | 3.2(2.48,4.13) | 3.15(88.77) |
| nausea | gastrointestinal disorders | 53(1.91) | 1.72(1.31,2.26) | 1.71(15.83) |
| acute kidney injury | renal and urinary disorders | 52(1.87) | 6.85(5.2,9.01) | 6.74(254.53) |
| off label use | injury, poisoning and procedural complications | 42(1.51) | 0.71(0.52,0.97) | 0.72(4.83) |
| urine albumin/creatinine ratio increased | investigations | 41(148) | 1738.99(1226.71,2465.2) | 1713.33(54615.16) |
| hypotension | vascular disorders | 40(1.44) | 5.09(3.73,6.96) | 5.03(129.55) |
| diarrhea | gastrointestinal disorders | 39(1.40) | 1.34(0.98,1.84) | 1.34(3.38) |
| hyponatremia | metabolism and nutrition disorders | 30(1.08) | 15.14(10.56,21.7) | 14.98(390.83) |
| fatigue | general disorders and administration site conditions | 30(1.08) | 0.83(0.58,1.19) | 0.83(1.04) |
| **JADER** |  |  |  |  |
| hyperkalemia | metabolism and nutrition disorders | 120(49.59) | 478.11(366.12,624.37) | 241.53(23221.57) |
| renal impairment | renal and urinary disorders | 33(13.64) | 11.89(8.22,17.19) | 10.4(281.27) |
| blood potassium increased | investigations | 15(6.20) | 242.79(136.55,431.71) | 227.8(2760.8) |
| blood creatinine increased | investigations | 9(3.72) | 41.98(21.29,82.75) | 40.45(333.18) |
| loss of consciousness | nervous system disorders | 4(1.65) | 5.17(1.92,13.92) | 5.1(13.16) |
| hepatic function abnormal | hepatobiliary disorders | 4(1.65) | 2.23(0.83,6) | 2.21(2.66) |
| glomerular filtration rate decreased | investigations | 3(1.24) | 21.43(6.78,67.71) | 21.18(56.51) |
| blood pressure decreased | investigations | 2(0.83) | 1.64(0.41,6.58) | 1.63(0.49) |
| cerebral infarction | nervous system disorders | 2(0.83) | 1.35(0.34,5.44) | 1.35(0.18) |
| interstitial lung disease | respiratory, thoracic and mediastinal disorders | 2(0.83) | 0.3(0.07,1.2) | 0.3(3.29) |
| myocardial infarction | cardiac disorders | 2(0.83) | 5.5(1.36,22.2) | 5.46(7.26) |
| malaise | general disorders and administration site conditions | 2(0.83) | 1.41(0.35,5.66) | 1.4(0.23) |
| bradycardia | cardiac disorders | 2(0.83) | 2.41(0.6,9.69) | 2.4(1.63) |
| renal failure | renal and urinary disorders | 2(0.83) | 6.01(1.49,24.26) | 5.97(8.23) |
| cardiac failure | cardiac disorders | 2(0.83) | 1.27(0.32,5.13) | 1.27(0.12) |
